# The Sick, the Churches, and the Hospitals

**Published:** 1887-11-05

**Authors:** Andrew Clark

**Affiliations:** President of the Hospitals Association


### The Sick, the Churches, and the Hospitals.

By Sir Andrew Clark, Bart., M.D., F.R.S., President of the Hospitals Association.

[We have pleasure in calling the attention of the clergy and ministers of religion of all denominations, managers of hospitals, boards of guardians, and all interested in the adequate support of hospitals and kindred charities to the following scheme, which would place those who have to visit the sick in their homes in a position to secure efficient help and advice in all cases of difficulty. Sir Andrew Clark has given much anxious consideration to the formulation of this scheme, and it is hoped that the clergy and ministers will take an early opportunity of drawing the attention of their congregations, and of the visitors amongst the poor in their parishes, to the facilities it affords them for making their work easier and more helpful to those they desire to benefit. Copies of the scheme and forms of application for members of associated local societies may be obtained on application to the Secretary of the Hospitals Association, Norfolk House, Norfolk Street, Strand, W.C. Some 1,500 copies in all of the scheme have been sent to the clergy and ministers in charge of places of worship within the metropolitan district. Correspondence and inquiries are cordially invited.]

One of the objects of the Hospitals Association is to afford precise information relative to the resources of the London and provincial hospitals; since it is admitted that delays, dangerous to health and life, are sometimes experienced in placing deserving cases in suitable institutions. To carry out this object, and also the other objects relating to the organisation, working, and maintenance of hospitals for which the Association was founded, it is necessary to secure the co-operation and association of all persons interested in the treatment of the sick poor, and especially of the managers of hospitals, boards of guardians, the clergy and ministers of all denominations, and local representative societies.

We therefore earnestly solicit your consideration of the following scheme of affiliation adopted by the Council of the Hospitals Association, which, if successful, will bring into communication all persons interested in dealing with the sick poor. This scheme will facilitate and enlarge the means of helping them, and will bring within the reach of every member of the Association an authentic and accurate knowledge of the economy and work of hospitals and of their claims to public support:

1. All persons interested in the treatment of the sick poor, especially the managers of hospitals, boards of guardians, the clergy and ministers of all denominations, and local representative societies, are invited to affiliate themselves to the Hospitals Association in London by the formation of a local Hospital Association of which at least one local medical practitioner should be a member.
2. The Hospitals Association will be the central and representative organisation, and its office in London will be a bureau of information, a medium of communication, and a centre of reference. Its aim will be to supply all kinds of information on hospital questions; to give advice respecting the organisation, management, and maintenance of all classes of hospitals; to arrange for lectures to such local Hospital Associations as may desire them; and to render assistance in the management of public meetings for the furtherance of hospital purposes.
3. Every affiliated local Hospital Association may nominate a representative, who will enjoy all the privileges of an individual member of the Association, and will be eligible for election to any of its honorary offices.
4. The inclusive annual subscription for each member, or for each affiliated local Association, will be one guinea per annum.
5. The new journal of the Association, The Hospital, which is published weekly, will be sent post free to each member of the Association, and to every representative of an affiliated local Association.

In committing this scheme to your favourable consideration, we desire to renew the expression of our conviction that affiliation to the Hospitals Association will greatly promote the interests of the sick poor, give much additional help to all who are concerned in caring for them, and contribute to the better understanding and the more liberal support of the great work carried on by our hospitals in the healing of the sick, the advancement of medical knowledge, and the dissemination of the experience so acquired for the common service of mankind. Any further information may be obtained on application to the Secretary of the Hospitals Association, Norfolk House, Norfolk Street, Strand, London, W.C.

